# Tight Evaluation of Real-Time Task Schedulability for Processor’s DVS and Nonvolatile Memory Allocation

**DOI:** 10.3390/mi10060371

**Published:** 2019-06-03

**Authors:** Sunhwa A. Nam, Kyungwoon Cho, Hyokyung Bahn

**Affiliations:** 1 Department of Computer Engineering, Ewha University, Seoul 03760, Korea; chunsun@ewha.ac.kr; 2 Embedded Software Research Center, Ewha University, Seoul 03760, Korea; cezanne@ewha.ac.kr

**Keywords:** real-time system, dynamic voltage scaling, task placement, low-power technique, nonvolatile memory

## Abstract

A power-saving approach for real-time systems that combines processor voltage scaling and task placement in hybrid memory is presented. The proposed approach incorporates the task’s memory placement problem between the DRAM (dynamic random access memory) and NVRAM (nonvolatile random access memory) into the task model of the processor’s voltage scaling and adopts power-saving techniques for processor and memory selectively without violating the deadline constraints. Unlike previous work, our model tightly evaluates the worst-case execution time of a task, considering the time delay that may overlap between the processor and memory, thereby reducing the power consumption of real-time systems by 18–88%.

## 1. Introduction

As IoT (internet-of-things) technologies grow rapidly for emerging applications such as smart living and health care, reducing power consumption in battery-based IoT devices becomes an important issue. An IoT device is a type of real-time system, of which, power-saving has been widely studied in terms of the processor’s dynamic voltage scaling (DVS). DVS lowers the supplied voltage of a processor when a load of tasks is less than the processor’s full capacity, thereby saving power consumption without violating the deadline constraints of real-time tasks. Although the execution time will increase due to the lowered supplied voltage, it would spend less power, as the power consumption in the CMOS (complementary metal-oxide semiconductor) digital circuits is proportional to the square of the supplied voltage [1]. 

Meanwhile, recent research has shown that memory subsystems are reaching a significant portion of power consumption in real-time embedded systems [2]. Such tremendous power consumption results mainly from the refresh operations of DRAM (dynamic random access memory) [2,3]. As DRAM is a volatile medium, it requires continuous power recharge in order to retain its data even in idle states. This article shows that the power consumption of real-time systems can be further reduced by combining a processor’s voltage scaling with hybrid memory technologies, consisting of DRAM and NVRAM. 

NVRAM (nonvolatile random access memory) technologies have emerged as an attempt of saving the power consumption of DRAM, as NVRAM does not need refresh operations [3]. NVRAM is byte-addressable memory similar to DRAM but it is better than DRAM in terms of energy-consumption and scalability. Thus, NVRAM is expected to be used as a main memory medium like DRAM in the not too far future [3,4,5]. Unfortunately, NVRAM has two critical weaknesses that prevent the total substitution of DRAM memory. First, the number of write operations allowed for each NVRAM cell is limited. For example, the current write endurance of PRAM (phase-change memory), a kind of representative NVRAM media, is known to be about 10^7^–10^8^ [3,6]. The second drawback is that the access time of NVRAM is expected to be slower than that of DRAM [5,7,8].

**Figure 1:**
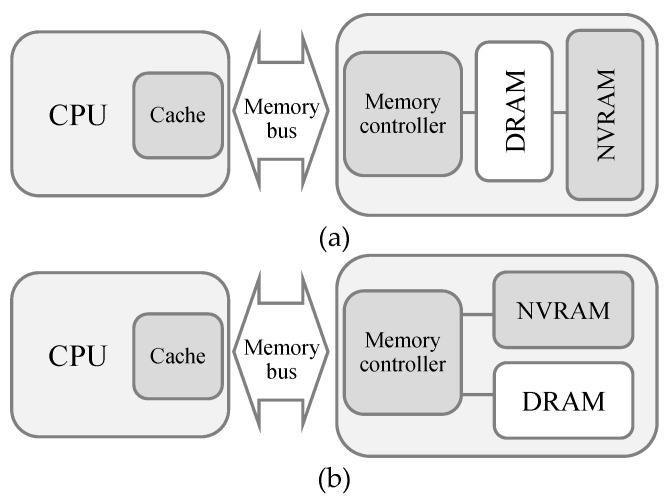
Architecture of the proposed system. (**a**) Hierarchical memory architecture, (**b**) hybrid memory architecture.

Despite these limitations, the prospect of NVRAM is still bright. One way of coping with the slow access latency and the write endurance problem of NVRAM is to adopt DRAM along with NVRAM [5,7]. This can hide the slow performance of NVRAM and also increase the lifespan of NVRAM. Two different memory architectures that comprise DRAM and NVRAM can be considered. The first architecture, depicted in Figure 1a, uses DRAM as an upper-level memory of NVRAM, which we call, the hierarchical memory architecture. The other memory architecture, depicted in Figure 1b, presents both DRAM and NVRAM at the same main memory level, managing them together under a single physical address space [5]. We call this architecture the hybrid memory architecture. In general-purpose time-sharing systems, the hierarchical memory architecture can improve the performance of virtual memory systems, as changing the backing store from slow HDD (hard disk drive) to fast NVRAM significantly reduces the page fault handling latency. However, as we focus on real-time systems, virtual memory is difficult to use, since page fault situations cannot be predicted beforehand, making the deadline guaranteed service difficult. This implies that the size of the DRAM should be large enough not to incur unexpected page faults, which is not fit for our target system, as we focus on reducing the use of DRAM for saving power consumption. Thus, we adopt the hybrid memory architecture and determine the location of tasks between DRAM and NVRAM in order to satisfy the deadline constraints by estimating the memory access latency beforehand. 

Although a task in NVRAM needs more time to be accessed, we can expect that an NVRAM resident task is still likely to be schedulable if it is executed under a low voltage mode of a processor. Our aim is to load tasks on NVRAM if it does not violate the deadline of real-time tasks, thereby reducing the power consumption further. To do so, we incorporate the task’s memory placement problem into the processor voltage scaling and evaluate the effectiveness of the unified approach. Simulation experiments show that our technique reduces the power consumption of real-time systems by 18–88%.

## 2. The Proposed Policy

Let *Γ* = {*τ*_1_, *τ*_2_, …, *τ_n_*} be the set of *n* independent tasks in a real-time system, which has a processor capable of dynamic voltage scaling, and main memory consisting of DRAM and NVRAM as shown in Figure 1b. Each task *τ_i_* is characterized by <*n_i_*, *CPI_i_*, *p_i_*, *s_i_*>, where *n_i_* is the number of instructions to be executed, *CPI_i_* is the clock cycles per instruction for *τ_i_*, *p_i_* is the period of *τ_i_*, and *s_i_* is the size of *τ_i_*’s memory reference stream during its execution. By considering common assumptions used in previous works [1], we make five assumptions for our system model.

A1. The size of the DRAM is large enough to accommodate entire task sets, but the power is turned off for the part of the DRAM where tasks are not loaded;
A2. Each task is executed independently and does not affect others;
A3. Context switch overhead (the overhead of switching a processor from one task to another) and voltage switching overhead (the overhead of switching the voltage mode of a processor from one to another) are negligible;
A4. The frequency of a processor is set to an appropriate level as the voltage supply is adjusted;
A5. We consider periodic tasks, and thus the period of a task implicitly determines the deadline of the task.

In our task model, the worst-case execution time (WCET) of a task can be determined based on the number of instructions to be executed in the processor and the memory access latency of the task. As modern embedded processors have an on-chip cache, main memory is accessed only upon a cache miss. Thus, memory delay caused by NVRAM also occurs only when a requested block is not in the on-chip cache. Once a block is loaded on the cache, then accessing a part of data within the block does not incur memory accesses. Let *c* be the cache block size and *s_i_* be the total size of memory reference stream in task *τ_i_*. Then, in the worst case, the number of memory accesses can be represented as *s_i_*/*c*. 

In our task model, WCET of a task is decided by the slower time component of executing instructions and accessing memory with the given voltage mode and the memory type. Specifically, WCET *t_i_* of a task *τ_i_* with the processor’s voltage level *v_i_* and the memory type *m_i_* is defined as:

*t_i_* = max{*t*_*i,*cpu_ (*v_i_, n_i_*), *t*_*i*,mem_ (*m_i_, s_i_*)} (1)

where *t_i,_*_cpu_ (*v_i_, n_i_*) is the execution time of *n_i_* instructions in the processor with the voltage level of *v_i_* and *t_i,_*_mem_ (*m_i_, s_i_*) is the memory access time of task *τ_i_* with the memory type *m_i_* and the size of reference stream *s_i_*, which can be subsequently defined as follows:

*t_i,_*_cpu_ (*v_i_, n_i_*) = (*n_i_*/*v_i_*) × *CPI_i_* × *LC* (2)

*t_i,mem_* (*m_i_,s_i_*) = (*s_i_*/*c*) × *LT*(*m_i_*) (3)

where *CPI_i_* represents clock cycles per instruction for the task *τ_i_*, *LC* is the cycle time, *c* is the cache block size, and *LT*(*m_i_*) is the memory access latency of the memory type *m_i_*. Note that the voltage level *v_i_* is set to 1 for the default voltage mode, and becomes less than 1 as the processor is set to a low voltage mode. 

The schedulability of a real-time task set *Γ* is tested by the utilization *U* of a processor as follows:

(4) $$U=\sum_{i=1}^{n}\frac{t_{i}}{p_{i}}\;\;\leq \;\;1$$

**Figure 2:**
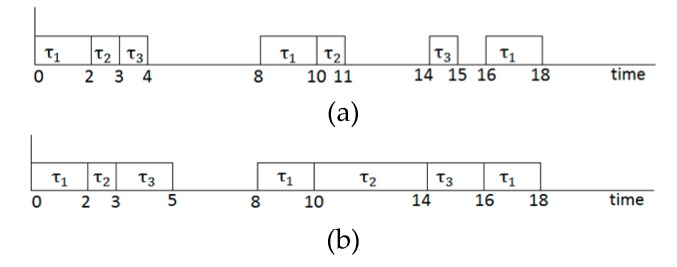
Comparison of the scheduled task set. (**a**) Scheduling result by original earliest deadline first (EDF), (**b**) scheduling result by the proposed approach.

We use the earliest deadline first (EDF) scheduling algorithm as it is known to perform scheduling without deadline misses, provided that there exist any feasible schedules on that task set [1]. Now, let us take a look at an example task set consisting of three tasks *τ*_1_, *τ*_2_, and *τ*_3_, whose worst-case execution times *t*_1_, *t*_2_, and *t*_3_ are 2, 1, and 1, respectively, under the default setting (i.e., normal voltage mode and DRAM only placement), and their periods are 8, 10, and 14, respectively. The schedulability of the task set is tested by calculating the utilization of the tasks *τ*_1_, *τ*_2_, and *τ*_3_, and adding up them i.e., *U* = 2/8 + 1/10 + 1/14 = 0.421. As the total utilization is less than 1, the task set is schedulable. Figure 2a shows the scheduling result for the task set with the EDF. Although the task set is schedulable, idle intervals reach up to 50% of the total possible working time of the processor. This inefficiency can be relieved by lowering the processor’s voltage for some idle intervals. For example, if two low voltage levels of 0.5 and 0.25 are applied for tasks *τ*_2_ and *τ*_3_, respectively, *t*_2_ and *t*_3_ will be 2 and 4, respectively. Accordingly, the utilization of the processor is increased to *U* = 2/8 + 2/10 + 4/14 = 0.736, which is still less than 1 and thus schedulable. Also, if we locate *τ*_3_ in NVRAM whose access latency is twice that of the DRAM, one may think that *t*_3_ will be 8, and thus it is not schedulable as *U* = 2/8+ 2/10 + 8/14 = 1.021 > 1. However, we tightly model WCET (worst case execution time) considering the overlapped time delay between the processor and memory, as shown in Equation (1), and thus *t*_3_ is still 4. Therefore, in our model, the utilization of the processor by applying both DVS and NVRAM becomes less than 1, still being schedulable. Figure 2b shows the scheduling result with our model when the aforementioned voltage scaling and memory mapping is adopted. As we see, idle intervals are decreased significantly when compared with the result in Figure 2a. 

As we deal with hard real-time systems, we assume that task scheduling is performed beforehand (i.e., off-line scheduling) and the scheduling does not change during the execution of the tasks. That is, the schedulability test is performed with the given resources (the voltage modes and memory types) at the design phase and the system resources are determined based on the schedulability test results, not to miss the deadlines of all tasks. This is a typical procedure for real-time task scheduling, and we extend it for memory placement. Note that as traditional real-time systems do not use virtual memory swapping due to the unpredictable page fault handling I/O (input/output) latency, the full address space of a task is loaded on the physical memory once it starts its execution. Thus, we also assume that the memory footprint of a task is determined at the scheduling phase, which is set to the maximum value for satisfying the deadline constraints in the worst case.

Algorithm 1 depicts the pseudo-code of our task setting and scheduling, of which the objective function is the maximization of *power_saving* with the constraint of *U* less than 1, implying that there are no deadline misses in the task set. Our problem can be modeled similar to the 0/1 knapsack problem. Thus, we solve the problem based on dynamic programming, which is one of the most efficient techniques to solve the 0/1 knapsack problem [9]. The algorithm tries to lower the power mode of each task *i* (1 ≤ *i* ≤ n) without exceeding the given utilization of each step. The state of each task with the utilization 1 will be our final solution. One can refer to the approximation algorithm of 0/1 knapsack problem for more details [9]. To decide the increment of utilization, we performed empirical analysis and found that the increment of 0.1 was appropriate in our case as the results were not sensitive when it became less than that value.

**Algorithm 1** Task setting and scheduling**Input:***n*, number of tasks; *power_saving*_(*i*, *U*)_, maximum power savings for tasks 1, 2, ..., *i* with utilization less than *U*; *power_saving_i_*, power savings obtained by adopting task *i* to low power mode; *U_i_*, increased utilization by adopting task *i* to low power mode **Output:** Φ, a schedule of all tasks  **for**
*i* is 1 to *n*
**do**  *power_saving*_(*i*, 0)_ ← 0; **end for** ** for**
*U* is 0.0 to 1.0 by 0.1 **do**  *power_saving*_(0, *U*)_ ← 0; **end for** **for**
*i* is 1 to *n*
**do**   **for**
*U* is 0.0 to 1.0 by 0.1 **do**    **if**
*U_i_* >*U*     *power_saving*_(*i, U*)_ ← *power_saving*_(*i*–1, *U*)_;   **else**     *power_saving*_(*i, U*)_ ← max{*power_saving*_(*i*–1, *U*)_,      power_saving _(*i*–1, *U* –*Ui*)_ + *power_saving_i_* };    **end if**  **end for** **end for**  set processor and memory states based on *power_saving*_(*n*, 1)_; schedule task set via EDF; 

## 3. Performance Evaluations

We compare our technique, called DVS-HM (dynamic voltage scaling with hybrid memory), with DVS-DRAM, HM (hybrid memory), and DRAM, which operate as follows.

- DVS-DRAM: This algorithm uses the processor’s dynamic voltage scaling, similar to DVS-HM, but does not use NVRAM and all tasks reside in DRAM;
- HM: This algorithm does not use the processor’s dynamic voltage scaling, but uses hybrid memory consisting of DRAM and NVRAM, and places tasks in NVRAM if it is still schedulable;
- DRAM: This is a baseline condition that does not adopt either the processor’s dynamic voltage scaling or hybrid memory technologies. That is, the processor is executed with its full voltage mode and all tasks reside in the DRAM.

The sizes of the DRAM and NVRAM are equally set to accommodate the entire task set. Table 1 shows the access latency and the power consumption of the DRAM and PRAM (phase-change random access memory), which is a type of NVRAM we experimented with. In theoretical aspects, there is no limitation in the level of the processor’s operating modes. However, as DVS-supported processors usually allow a very limited number of operating modes for practical reasons, we also allow four voltage levels of 1, 0.5, 0.25, and 0.125. 

Power consumption in the memory system can be divided into active and idle power consumption. Idle power consumption includes the leakage power and refresh power. The leakage power is power consumed even when the memory is idle and the leakage power of NVRAM is negligible compared to that of DRAM. DRAM memory cells store data in small capacitors that lose their charge over time and must be recharged. This process is called refresh. Regardless of the read and write operations, DRAM consumes considerable refresh power to sustain refresh cycles to retain its data. However, this is not required in NVRAM because of its non-volatile characteristics. Active power consumption, on the other hand, refers to the power dissipated when data is being read and written. In our experiments, power consumptions of processor and memory are separately evaluated and then accumulated. The total power consumption *Power_total_* is evaluated as:

*Power*_total_ = *Power*_cpu_ + *Power*_mem_ (5)

where:

*Power*_cpu_*=* ∑_cpu_mode_ {*Unit_Power*_cpu_mode_ × *Cycles*_cpu_mode_} (6)

and:

*Power*_mem_ = ∑_mem_type_ {*Unit_Active_Power*_mem_type_ × *Active_Cycles*_mem_type_ + *Unit_Idle_Power*_mem_type_ × *Idle_Cycles*_mem_type_}. (7)

*Unit_Power*_cpu_mode_ is the unit power consumption per cycle for the given CPU mode and *Cycles*_cpu_mode_ is the number of CPU cycles with the given CPU mode. *Unit_Active_Power*_mem_type_ is the active power per cycle for accessing the given memory type, *Active_Cycles*_mem_type_ is the number of memory cycles for accessing the given memory type. *Unit_Idle_Power*_mem_type_ is the static power per cycle, including both the leakage power and refresh power for the given memory type, and *Idle_Cycles*_mem_type_ is the number of memory cycles for the idle period for the given memory type.

We performed experiments under both synthetic and realistic workload conditions. In the synthetic workload, we created 10 task sets varying the load of tasks for a given processor capacity, similar to previous studies [10]. In the case of the realistic workload, we used two workloads, the robotic highway safety marker (RSM) workload [11] and the IoT workload [12]. Table 2 and Table 3 list the workload configurations for the RSM and IoT workloads that we experimented with [11,12].

### 3.1. Experiments with Synthetic Workloads

**Figure 3:**
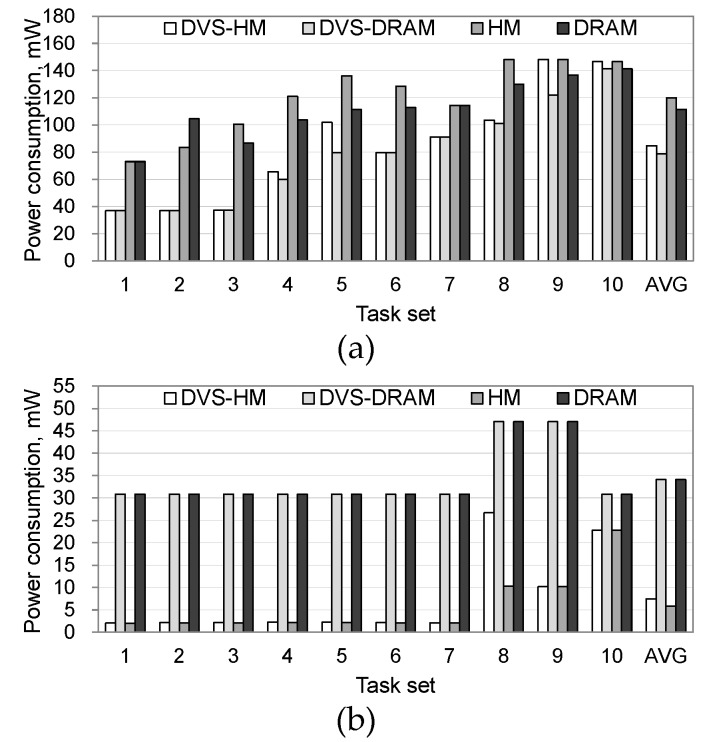
Processor and memory power consumption under synthetic workloads. (**a**) Power consumption in processor, (**b**) power consumption in memory. DVS-HM = dynamic voltage scaling with hybrid memory; DVS-DRAM = dynamic voltage scaling with dynamic random access memory; HM = hybrid memory; DRAM = dynamic random access memory.

Figure 3 shows the power consumption in processor and memory for the four schemes when the synthetic workload is used. As shown in Figure 3a, DVS-DRAM and DVS-HM, which adopt voltage scaling, similarly saved a substantial amount of processor’s power consumption. HM and DRAM, which do not use DVS, showed a relatively higher power consumption than DVS-DRAM and DVS-HM, although the gap became small in some cases. In particular, DVS was less effective as a task set’s load approached the full capacity of a processor. This was because the chance of utilizing idle periods of a processor by DVS becomes difficult in such cases. Note that the load of a task set became heavy as the task set number increases in our cases. 

Figure 3b shows the power consumption in the memory. The DVS-HM and HM, which use NVRAM along with DRAM, consumed less energy than the DVS-DRAM and DRAM, which only use DRAM. This is because the idle power of NVRAM is close to zero, and thus the reduced size of DRAM—by adopting NVRAM—saved the refresh power of the DRAM. However, as the latency of NVRAM is longer than that of DRAM, executing a task in NVRAM may increase the execution time in the processor, possibly increasing the processor’s power consumption. However, as shown in Figure 3a, such a phenomenon happened only in HM and it disappeared in DVS-HM, which uses voltage scaling along with hybrid memory placement. When comparing the DVS-DRAM and DVS-HM, we can see that adopting NVRAM does not increase the processor’s power consumption if DVS is used. This is because power-saving can be maximized by executing a processor in a low voltage mode when the task is located in NVRAM.

**Figure 4:**
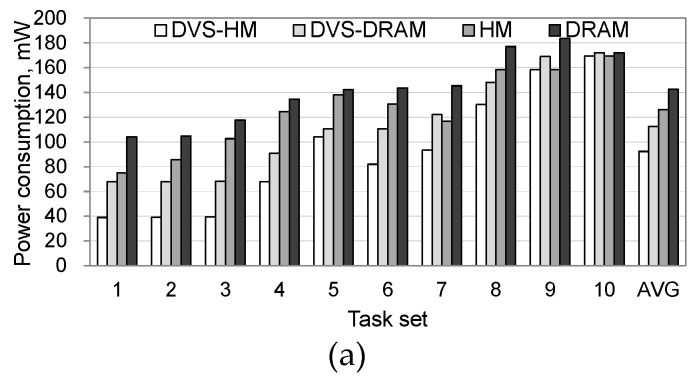

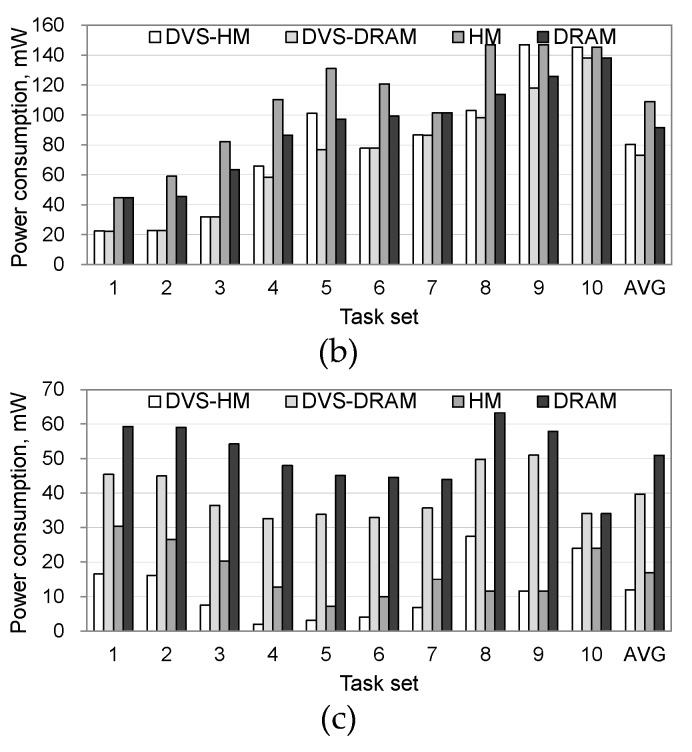
Power consumptions under synthetic workloads. (**a**) Total power consumptions, (**b**) active power consumptions, (**c**) idle power consumptions.

**Figure 5:**
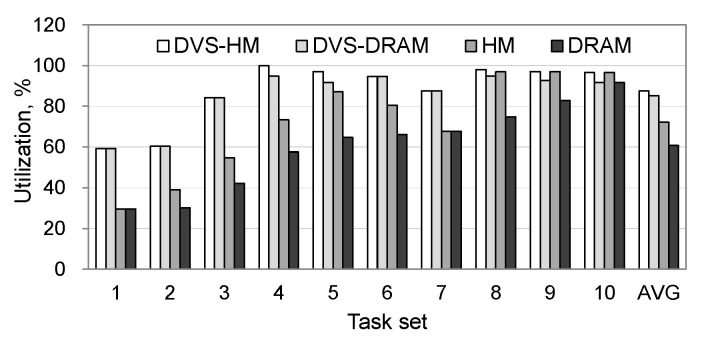
Processor utilizations under synthetic workloads.

Figure 4a shows the total energy consumption by adding up the consumed energy in processor and memory. DVS-HM saved the energy consumption of the DRAM, DVS-DRAM, and HM by 36%, 18%, and 28%, respectively. Figure 4b,c separately show the active and idle power consumptions. Although the DVS-HM performed worse than the DVS-DRAM, in terms of active power consumption, it performed the best in idle power consumption, leading to the minimized total power consumption. Figure 5 shows the processor’s utilization. As we see, DVS-HM showed the highest utilization in all cases and was close to 100% in some cases.

### 3.2. Experiments with Realistic Workloads 

**Figure 6:**
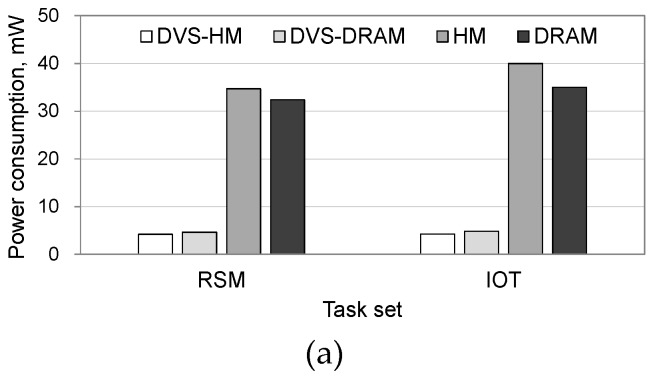

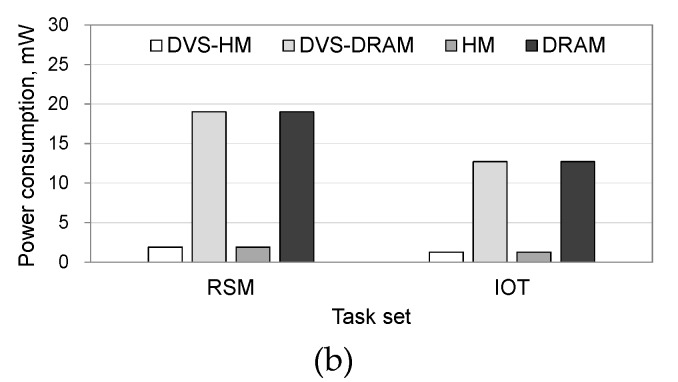
Processor and memory power consumptions under realistic workloads. (**a**) Power consumption in processor, (**b**) power consumption in memory.

To see the effectiveness of the proposed algorithm in more realistic situations, we performed additional experiments under two realistic workload conditions, a robotic highway safety marker (RSM) workload [11] and an IoT workload [12]. Similar to the synthetic workload cases, we show that the proposed algorithm is effective in increasing the processor’s utilization and decreasing the power consumption. Figure 6 shows the power consumptions in processor and memory separately when the RSM and IoT workloads are used. For both workloads, power consumption in the processor was significantly reduced when the DVS was used. Specifically, DVS-HM and DVS-DRAM saved the processor’s power consumption by 86–88% in comparison with HM and DRAM, as shown in Figure 6a. 

When we compared the power consumption in memory, algorithms that use NVRAM along with DRAM significantly reduced the power consumption, as shown in Figure 6b. Specifically, the DVS-HM and HM consumed 89–90% less power than the DVS-DRAM and DRAM, which only use DRAM. This is because the idle power of NVRAM is very small. 

**Figure 7:**
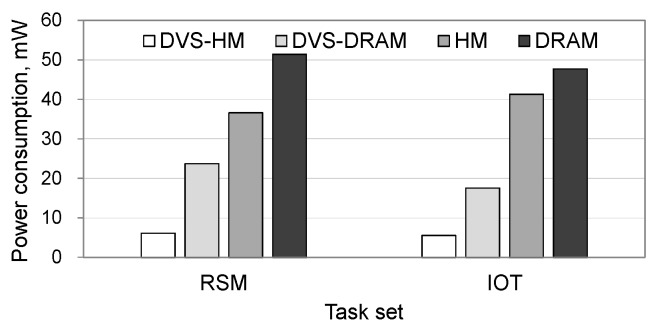
Total power consumption under realistic workloads.

**Figure 8:**
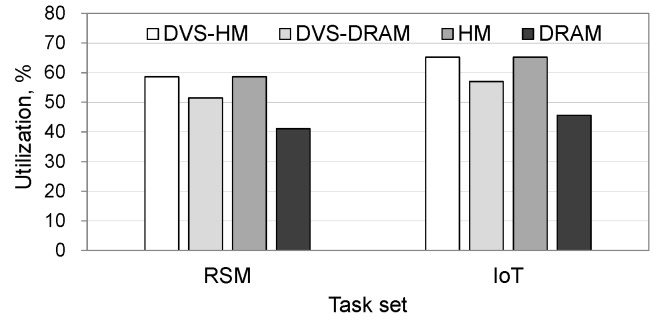
Processor utilizations under realistic workloads.

Figure 7 shows the total power consumption when realistic workloads are used. As shown in the figure, the trends of the graphs are consistent with the synthetic workload cases. Specifically, DVS-HM saved the power consumption of DRAM, DVS-DRAM, and HM by 88%, 74%, and 83%, respectively, in the RSM workload and 88%, 68%, and 87%, respectively, in the IoT workload. Figure 8 shows the processor’s utilization for realistic workloads. As can be seen from the figure, DVS-HM exhibited the highest utilization by adopting low-power resource configurations in both the processor and memory. The HM also showed a high utilization similar to DVS-HM, but this was not due to the low voltage setting of the processor, as HM does not use DVS. In fact, the high utilization of the HM was caused by the stalls in executing the instructions while accessing the slow NVRAM memory. Due to this reason, the HM presented a significantly larger power consumption in the processor, although its utilization became high, which was different from the DVS-HM cases. 

## 4. Related Works

### 4.1. Hybrid Memory Technologies

Recently, hybrid memory technologies consisting of DRAM and NVRAM have been catching interest. As NVRAM is byte-accessible, similar to DRAM, but consumes less energy and provides higher scalability than DRAM, it is anticipated to be adopted in the main memory hierarchy of future computer systems. Mogul et al. suggest an efficient memory management policy for DRAM and PRAM hybrid memory [4]. Their policy tries to place read-only pages in PRAM, while writable pages in DRAM, thereby reducing the slow PRAM writes [4]. Dhiman et al. propose a hybrid memory architecture consisting of PRAM and DRAM, which dynamically moves data between PRAM and DRAM in order to balance the write count of PRAM [5]. Qureshi et al. propose a hierarchical memory architecture consisting of DRAM and PRAM [7]. Specifically, they use DRAM as the write buffer of PRAM in order to prolong the lifespan of PRAM and hide the slow write performances of PRAM. Lee et al. propose the CLOCK-DWF (clock with dirty bits and write frequency) policy for hybrid memory architecture, consisting of DRAM and PRAM [6]. They allocate read-intensive pages to PRAM and write-intensive pages to DRAM by online characterization of memory access patterns. Zhou et al. propose a hierarchical memory architecture consisting of DRAM and PRAM [8]. In particular, they propose a page replacement policy that tries to reduce both the cache misses and the write-backs from DRAM. Narayan et al. propose a page allocation approach for hybrid memory architectures at the memory object level [13]. They characterize memory objects and allocate them to their best-fit memory module to improve performance and energy efficiency. Kannan et al. propose heterogeneous memory management in virtualized systems [14]. They designed a heterogeneity-aware guest operating system (OS), which allows for placing data in the appropriate memory, which avoids page migrations. They also present migration policies for performance-critical pages and memory sharing policies for guest machines. 

### 4.2. Low-Power Techniques for Real-time Scheduling

Many studies have been performed on DVS in order to reduce power consumption in real-time systems [15,16,17,18]. Pillai and Shin propose a mechanism of selecting the lowest operating frequency that will meet deadlines for a given task set [19]. They propose three algorithms for DVS: Static DVS, cycle-conserving DVS, and look-ahead DVS. Static DVS selects the voltage of a processor statically, whereas cycle-conserving DVS uses reclaimed cycles for lowering the voltage when the actual execution time of a task is shorter than the worst-case execution time. Look-ahead DVS lowers the voltage further by determining future computation requirements and deferring the execution of the task in accordance. Lee et al. use the slack time to lower the processor’s voltage [1]. Specifically, initial voltages can be dynamically switched upon reclaiming unused clock cycles when a task completes before its deadline. Lin et al. point out that there is a memory mapping problem, as heterogeneous memory types are used [10]. They use dynamic programming and greedy approximation for solving the problem. Zhang et al. propose task placement in hybrid memory to save energy consumption [20]. In their scheme, tasks are located one by one in the NVRAM and the schedulability is checked. This procedure is repeated until the locations of all tasks are determined. Ghor and Aggoune propose a slack-based method to find the least voltage schedule for real-time tasks [16]. They stretch the execution time of tasks through off-line computing and schedule tasks as late as possible without missing their deadlines.

## 5. Conclusions

This article presented a new real-time task scheduling approach that unifies the processor’s voltage scaling and task placement in hybrid memory. Our approach incorporates the task placement in hybrid memory into the task model of voltage scaling in order to maximize the power-saving of real-time systems. The experimental results showed that the proposed technique reduces the power consumption of real-time systems by 18–88%. In the future, we will perform measurement studies in real systems in order to assess the effectiveness of the proposed approach in more realistic situations.

## Figures and Tables

**Table 1:** DRAM (dynamic random access memory) and NVRAM (nonvolatile random access memory) characteristics.

Characteristics	DRAM	PRAM
Read latency	50 (ns)	100 (ns)
Write latency	50 (ns)	350 (ns)
Read energy	0.1 (nJ/bit)	0.2 (nJ/bit)
Write energy	0.1 (nJ/bit)	1.0 (nJ/bit)
Idle power	1 (W/GB)	0.1 (W/GB)

**Table 2:** Robotic highway safety marker (RSM) task set parameters. WCET = worst case execution time; PID = process id.

Task	Period	WCET
Serial	7.8125 ms	100 μs
Length	7.8125 ms	1 ms
Way Point	23.4375 ms	2.5 ms
Encoder	23.4375 ms	350 μs
PID	23.4375 ms	1.06 ms
Motor	23.4375 ms	250 μs

**Table 3:** Internet-of-things (IoT) task set parameters. WCET = worst case execution time; GUI = graphical user interface.

Task	Period	WCET
Sense Temperature	100 ms	10 μs
Send data to server	1 min	6 ms
Sense Vibration	10 ms	600 μs
Compress and send	1 s	7.5 ms
Get info. & calc.	10 ms	1 ms
Control machine	10 ms	1 ms
Update GUI	1 s	20 ms
